# Using multi-scale behavioral investigations to inform wild pig (*Sus scrofa*) population management

**DOI:** 10.1371/journal.pone.0228705

**Published:** 2020-02-07

**Authors:** Jennifer L. Froehly, Nathan R. Beane, Darrell E. Evans, Kevin E. Cagle, David S. Jachowski

**Affiliations:** 1 Department of Forestry and Natural Resources, Clemson University, Clemson, South Carolina, United States of America; 2 Environmental Laboratory, US Army Engineer Research and Development Center (ERDC), Vicksburg, Mississippi, United States of America; 3 Natural Resources Management Branch, Fort Hood Directorate of Public Works, Fort Hood, Texas, United States of America; Universitat Autonoma de Barcelona, SPAIN

## Abstract

Assessing invasive species ecology at multiple scales is needed to understand how to focus ecological monitoring and population control. As a widespread invasive species, wild pigs (*Sus scrofa*) frequently disrupt land management programs. We provide a detailed, multi-scaled view of the behavior of wild pigs at Fort Hood, Texas, USA by assessing seasonal and daily movement patterns, and diet. First, we quantified movement behavior through assessment of both seasonal home range size and first passage time movement behavior from 16 GPS-collared wild pigs. Home ranges were relatively large (mean: 10.472 km^2^, SD: 0.472 km^2^), and Cox proportional hazard models predicted that pigs moved slowest at temperature extremes (15< °C <30), in the spring, in rougher terrain, and in grassland communities. Secondly, we analyzed wild pig stomach contents to determine diet throughout the year. Diet was primarily plant-based, and showed seasonal variation in such items as hard and soft mast, and the foliage of forbs and woody plants. Integration of both movement and diet analyses indicate that wild pigs are more likely to be lured into baited traps in the winter when movement rates are highest and plant-based food resources are likely less abundant. Wild pigs are likely to have the greatest impact on vegetative communities in grassland habitats during the spring season when movement is restricted. Collectively, this multi-scaled approach provided detailed information on wild pig behavioral ecology in this area that would not have been apparent by looking at any single measure individually or only at a large spatial scale (i.e., home range), and could be a useful approach in other invasive species management programs.

## Introduction

Invasive animal populations can be inherently difficult to manage due to a lack of natural predators, high reproductive rates, and adaptability [1]. When a species is highly plastic and can occur in numerous ecosystems, site-specific management can help to exploit weaknesses in the presence of specific biotic and abiotic factors [2, 3]. Site-specific management requires knowledge of the local environment and how the invasive species interacts within this environment. Since these interactions can occur at multiple spatial scales within an environment [4], using multi-scaled research approaches to investigate invasive species ecology can help managers better identify effective control techniques and assess impacts to the invaded environment [5].

**Fig 1:**
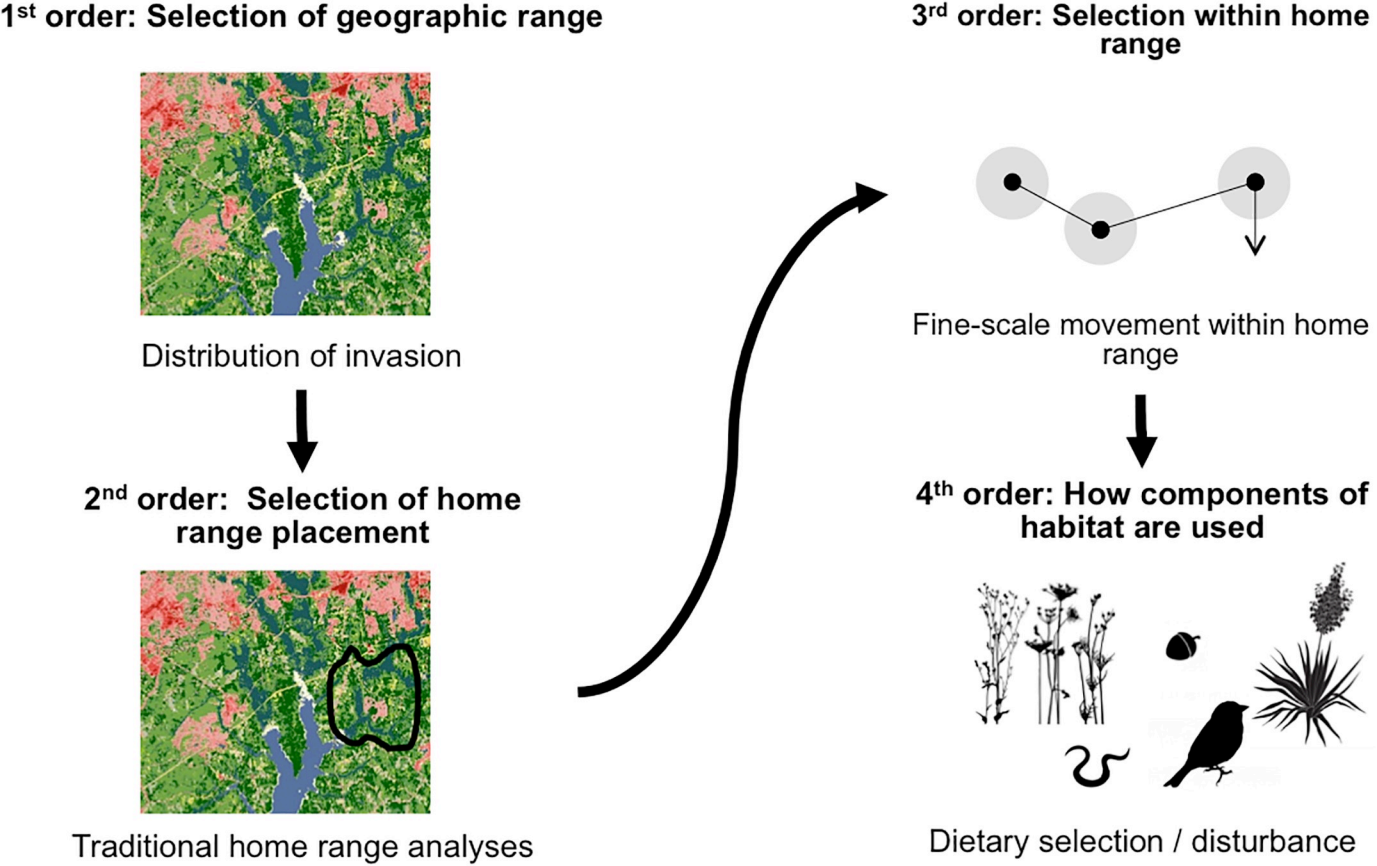
Diagram based on Johnson (1980) [6], indicating four scales of resource selection and associated application to invasive species ecology. At the first order, species distribution (green) can be identified at the landscape scale, compared to second order selection where the location of an individual’s home range is known within the landscape. Third order selection can determine selection within the home range based on fine-scale movement behavior, and fourth order selection involves investigations into how invasive species use or disturb habitats at the finest scale.

Applying current animal spatial ecology and habitat selection theory to invasive species could be particularly valuable in improving our multi-scaled understanding of their ecology and guiding management strategies. Johnson (1980) proposed that habitat selection is a hierarchical process by which a species makes decisions that occurs on four spatial scale or orders, ranging for where the species selects it range, to at the finest scale, how it utilizes individual components of habitat (Fig 1)[6]. While studies of invasive species commonly assess one, or perhaps two, of these scales individually [e.g., 7,8], researchers seldom attempt to measure habitat use across ≥2 scales simultaneously [but see 9]. Such nuanced understanding could be particularly valuable when there is clear evidence that at a coarse-scale a species is seemingly not limited or widely distributed across the landscape (i.e., first order selection), and current management efforts are failing to control invasive populations.

Wild pigs (*Sus scrofa*), native to Eurasia, are now a widespread invasive species in Australia, Africa, and the Americas. Their rooting behavior destroys plant material and can alter ecosystems by affecting soil processes, structure, and biota, as well as altering plant communities and regeneration cycles [10]. In the United States, wild pigs currently occur in 38 states [11] and cause over 1 billion dollars in damage to agricultural crops and infrastructure annually [12].

Management of invasive wild pig populations is difficult due to prolific breeding, high survivorship, and the absence of natural predators [13]. Information on site-specific movement and habitat preference can inform managers when and where pigs are causing the largest ecological impacts [14]. Traditionally, these relationships have been assessed at the home-range scale [15]. However, more recent use of GPS collars provides the opportunity to assess movement paths and fine-scale movement behaviors within the home range [16, 17, 18]. To identify the direct impacts of foraging activities at perhaps the finest behavioral scale, it is necessary to analyze dietary preferences [19]. Collectively, understanding habitat use across each of these scales allows for a more complete picture of wild pig behavior and potential impacts, and can be used to identify where the animals would be easiest to trap at particular times of the year.

In Texas, since their introduction by colonists in the early 1800s, wild pigs have dispersed to every county, making them a ubiquitous concern for land management across the state. Wild pig behavior is particularly a concern at the Fort Hood Military Installation (hereafter Fort Hood) because it threatens the efficacy of both military use and federally mandated natural resource management [20]. Fort Hood is home to populations of the federally endangered golden-cheeked warbler (*Dendroica chrysoparia*) and the recently delisted black-capped vireo (*Vireo atricapilla*), providing an impetus for population management of wild pigs as pig foraging behavior could destroy critical habitat of these protected species [20]. Site-specific investigations of wild pigs on Fort Hood are needed to direct monitoring and management efforts, particularly those that assess both intensity of use within Fort Hood habitats, and the direct impact they are having through foraging.

In this study, we analyzed wild pig movement, habitat use, and diet to simultaneously assess wild pig habitat use patterns across three scales (Fig 1). Our specific objectives were (1) to calculate seasonal home ranges (2) to identify biotic and abiotic factors that drive daily movement and habitat use, and (3) to determine the seasonal variation in diet. More broadly, our study provides one of the first examples of how multi-scale (i.e., >2) assessments of invasive species habitat use behavior can be used to guide future ecological monitoring and control strategies to mitigate the impacts of this invasive species.

## Methods

### Ethics statement

Fort Hood veterinarians were present during handling, transporting and immobilization of wild pigs in this study. All actions and methods involving animals, including direct take through hunting and trapping, adhered to protocols established and approved by US Army Engineer Research and Development Center IACUC field protocol (EL-FRP-2016-2).

### Study area

This study took place on Fort Hood in Killeen, Texas. The military installation encompasses 88,390 hectares and was established in 1942. Historic land cover prior to establishment of Fort Hood was a mixture of grasslands, oak savannahs, shrubland, oak-juniper forests, and riparian corridors. Current land cover is comprised of 33.61% ruderal, 26.62% woodland, 22.54% juniper, 10.91% grassland, 5.66% shrubland, 0.3% water, and 0.27% riparian (Fort Hood vegetation classification layer, The Nature Conservancy 2008). Elevation of the study area ranged from 177.0–377.2 m. Approximately 90% of Fort Hood is used for training and almost 30% is reserved for live fire [20]. Access to live-fire training areas is restricted to people, which limits management activities. However, this area are accessible to wild pigs as the area is only fenced using standard 3- or 4-strand barbed wire cattle fencing. The climate is warm and temperate with an official Köppen climate classification of humid subtropical. During our study, daily average temperatures were 12 °C, 20 °C, 28 °C, and 20 °C for winter, spring, summer, and fall respectively. These temperatures were 1–4 °C higher than the 1981–2010 climate normal’s [21]. Yearly precipitation was 760 mm, 50 mm lower than the 1981–2010 normal [21]. Seasonal precipitation was higher in the winter and spring, and lower in the summer and fall when compared with the 1981–2010 seasonal precipitation normal’s [21].

### Data collection

#### Movement

We captured wild pigs using corral traps from December 2016 to June 2017. Our goal was to capture 12 individuals from different sounders (family groups) over the course of the study. The first pig was collared on 10 December 2016, and trapping occurred opportunistically until 21 June 2017. Adult individuals (weighing > 28 kg) were selected from each sounder and outfitted with a Telonics^®^ GPS/Iridium radio-collar. We programmed GPS collars to record a position fix and temperature every three hours. Location data were obtained until 31 December 2017 (Table 1).

**Table 1:** Timeline for each pig equipped with a GPS collar. Number of GPS locations acquired for each pig were tallied by season on Fort Hood Military Installation 2016–2017.

INDIVIDUAL	START GPS	END GPS	SEX	Number GPS fixes acquired					% Fixes Unsuccessful
				Fall	Spring	Summer	Winter	Total	
PIG01	12/10/16	1/20/17	M				326	326	7.2
PIG02	12/10/16	3/10/17	F		74		638	712	14.5
PIG03	1/13/17	3/4/17	F		28		373	401	0.6
PIG04	1/13/17	3/22/17	F		173		365	538	2.0
PIG05	12/10/16	6/12/17	M		726	86	642	1454	40.4
PIG06	12/15/16	6/5/17	F		733	36	600	1369	1.0
PIG07	2/10/17	8/16/17	F		726	610	148	1484	2.5
PIG08	4/19/17	12/31/17	F	718	330	711	247	2006	2.1
PIG09	4/20/17	12/31/17	F	654	242	695	222	1813	0.7
PIG10	6/21/17	12/31/17	F	701		558	247	1506	10.4
PIG11	12/15/16	12/31/17	M	663	647	601	842	2753	0.9
PIG12	2/10/17	11/8/17	M	53	705	462	136	1356	9.6
PIG12	4/19/17	12/31/17	F	674	291	664	206	1835	2.5
PIG14	5/23/17	12/31/17	M	351	59	445	200	1055	0.7
PIG15	5/23/17	12/31/17	F	721	62	702	246	1731	0.3
PIG16	4/19/17	12/31/17	F	644	278	645	185	1752	5.7

#### Diet

From December 2016 through October 2017, Fort Hood personnel and U.S. Army Engineer Research and Development Center biologists collected wild pigs seasonally during controlled hunting and in corral traps. Lone adult individuals were targeted when possible, and no more than three individuals were collected from the same sounder. Animals were weighed and sexed: individuals weighing more than 28 kg were classified as adults and considered for inclusion in the food habitat portion of the study. No sub-adults or juveniles were used in the diet analysis. Eighty-eight animals (46 males and 42 females) met our size criteria and were included in the seasonal food habit analysis. Stomachs were removed in the field, labeled according to sex, given a unique identification number, and frozen solid within 3–4 hrs. We thawed Stomachs to room temperature in the lab and thoroughly mixed to homogenize the contents. We collected a 0.5 L sample from each stomach for the gross analysis. We washed samples at low pressure through a 6.3 mm mesh sieve to remove fine, unidentifiable material and dirt [22, 23]. During our gross analysis we did not examine material that was washed through the sieve. We visually identified food items retained in the sieve to the lowest taxon possible and placed items into the following major categories: graminoids, forb and woody foliage, cactus, roots/bulbs/tubers, corn, fruit/soft mast, hard mast, invertebrates, vertebrates, fungi, trash and unidentifiable items. We measured the volume of all food items classified in stomach samples to the nearest 0.5 ml using volumetric displacement [24].

### Data analysis

#### Movement

For each individual, we analyzed movement at both the seasonal home range and daily movement scales. GPS data were only included in analysis after each pig had settled following its release after collaring. Conservatively, this took up to 48 hrs post-release. Therefore, we censured all locations prior to 48 hrs post-release from further analysis. Any attempted but unsuccessful GPS fixes were also removed (Table 1). For our home range analysis, we separated data into seasons and calculated home ranges for each season(s) in which the data for an individual lasted at least one-half of the season. Based on seasonal climatic patterns in this region, we designated winter as 1 December through 28 February; spring as 1 March through 31 May; summer as 1 June through 31 August; and fall as 1 September through 30 November [25].

To evaluate our first objective, we estimated home range for each individual by calculating both 95% and 50% kernel density estimates [26] with a plug-in smoothing factor [27]. We used home range estimates to determine average 95% home range size by season and 50% core area by season for each individual. We performed a linear mixed model ANOVA for each 95% and 50% home range using lme4 [28] in Program R version 3.3.3 [29] to test for differences in seasonal home range size and sex differences in home range, including a random effect of individual sampled. If the resulting p-value was <0.05, we performed a post hoc Tukey’s pairwise comparison test to determine which seasons were different from each other.

To confirm that hourly movement followed a crepuscular pattern wild pigs are known to exhibit, we calculated average step length between each three-hour GPS fix. We then used a first-passage time (FPT) analysis approach to assess our objective of analyzing resource use and movement on a daily scale. First-passage time is an area-restricted search metric measuring how long it takes an individual to cross a circle of a given radius and is calculated along regular steps of a trajectory [30]. We cut individual trajectories into daily trajectories and used the adehabitatLT package [31] in Program R version 3.3.3 [29] to analyze FPT of interpolated points every 60 m along each daily trajectory with radii from 30 m to 15,000 m by 30 m increments. We initially selected radii in 30 m increments up to 15,000 m because 30 m was the average positional error of a stationary collar plus two standard deviations [32], and 15,000 m was just under the average 95% KDE home range radius (see above) if circular home ranges were assumed. We then calculated the variance of the log FPT values for each daily trajectory and averaged daily trajectory variances for each individual. The population log FPT variance was calculated by averaging the individual average variances and used the mean maximum variance of the population to identify what FPT radius values should be used as the response variable in further modeling [30]. From these methods, we identified 30 m as the radius with the population mean maximum variance and the scale at which area-restricted search was occurring.

We extracted 30 m FPT values and habitat covariates every 60 m along the full trajectory of each individual. We used a 1 m digital elevation model (DEM; Fort Hood DPW Environmental Natural Resources Management Branch 2016) to create a topographic roughness index (TRI) [33] raster using the terrain function in the R raster package and extracted the mean TRI for each 30 m radius first passage time circle. We used the Fort Hood streams layer (Fort Hood DPW Environmental Natural Resources Management Branch 2016) to extract the distance (m) to the nearest year-round/perennial water source from each FPT centroid. Ephemeral or intermittent water sources were not included in this analysis given the uncertainty with which these sources were actually available to animals during any given period during our year-long study. We used the Fort Hood vegetation classification layer (The Nature Conservancy 2008) to extract generalized land cover categorical variables that represented the dominant land cover within the 30m radius FPT circles. Landcover types included grassland, juniper, woodland, ruderal, shrubland, riparian, and water. We used time stamps and temperature data from each locational fix to interpolate season (defined above) and collar temperature for each FPT circle.

We created seven *a priori* models reflecting how we hypothesized that biotic and abiotic covariates influenced first passage time, and thus resource use and movement rates (Table 2). We included a model for each singular covariate of mean topographic roughness index, distance to year-round water, temperature, landcover type, and season, as well as a model with an additive effect of landcover and distance to water, and a global model that included all covariates. We predicted that pigs would remain in areas with low topographic roughness longer and move quickly through areas of high topographic roughness because rougher areas are harder to maneuver in [34]. We predicted that pigs would remain in areas closer to water longer and move quickly through areas farther from water due to the use of water by pigs to thermoregulate [35]. We predicted that pigs would be more stationary when temperatures were at their extremes and would be more mobile when temperatures were moderate due to thermoregulation needs [11, 35, 36]. Thus, we used a quadratic term of temperature in our models. We hypothesized that pigs would spend more time in preferred habitat, which would provide their needs for cover, food resources, and water [13, 37]; leading us to predict that woodland, riparian, juniper, and shrubland habitats would be preferred over ruderal and grassland habitats. Grassland was used as the base/reference category. We also hypothesized that movement rates would differ seasonally due to differences in temperature, food resources and breeding cycle [38]. Pigs were predicted to move more in fall and winter and less in spring and summer due to high temperatures, abundance of food resources, and the higher likelihood of piglets being present in the spring and summer [10]. Fall was used as the base/reference category.

**Table 2:** *A priori* models of covariates hypothesized to influence first passage time (FPT) and their predicted effects on GPS collared wild pigs on Fort Hood Military Installation in 2016–2017.

*a priori* covariate structure	prediction
Topographic roughness index (TRI)	FPT increases with decreasing TRI
Distance to water	FPT increases with decreasing distance to water
Temperature^2^	FPT increases at temperature extremes
Landcover type	FPT increases in woodland, juniper, riparian, and shrubland, FPT decreases in ruderal and grassland
Season	FPT increases in spring and summer and decreases in fall and winter
Landcover type + distance to water	FPT increases in woodland, juniper, riparian, and shrubland, FPT decreases in ruderal and grassland, and FPT increases with decreasing distance to water
Global	All of the above predictions contribute to FPT

Because we were using continuous time-to-event data (FPT), we used Cox proportional hazard models with individual frailty (random effect) [39], using a survival object comparing FPT time to event (leaving the circle) as the response variable in the Survival package in Program R. We used penalized log likelihoods and penalized degrees of freedom terms to calculate Akaike Information Criterion (AIC) [40, 41], and interpreted models that contributed to the 90% model weight. Effects of the top model(s) were analyzed using hazard ratios (exp^β^), where a hazard ratio for a continuous variable >1 means that the hazard increases with the variable, causing a decrease in FPTs, and a hazard ratio <1 means that the hazard is decreasing with the variable, causing an increase in FPT [39]. Categorical variable hazard ratios are in reference to the base category where a hazard ratio > 1 means FPT are lower comparative to the base category, and a hazard ratio < 1 means FPT are higher comparative to the base category [42]. Covariates had a significant effect on FPT when the hazard ratio 95% confidence interval did not overlap one [43]. In order to relate hazard ratios and FPT to resource use preference, we assumed that higher FPT indicated a preferred resource where an individual would spend more time.

#### Diet

For each season, we calculated the frequency with which dietary items were detected at least once in an individual as well as the percent of volume each item represented in a stomach sample. We calculated the percent volume of each food category for each individual by dividing a given category by the summed weight of identified food items within a given season. We also calculated frequency of occurrence for each food item as the number of individuals with a specified food type divided by the total number of stomachs.

We conducted a one-way analysis of variance (ANOVA) to test our hypothesis that the frequency of wild pig dietary items differed seasonally. Specifically, we predicted that frequency of consumption of forbs, fruit and soft mass production would be highest in spring and summer, and that consumption of hard mast would be highest in fall and winter [23, 44]. If the ANOVA showed that the mean for at least one season was different (*p* > 0.05), a post hoc Tukey’s pairwise analysis was run to determine which means differed from each other. We evaluated all measures for normality prior to analysis, conducted transformations where appropriate, and evaluated significance when α ≤ 0.05.

## Results

### Movement and habitat

**Fig 2:**
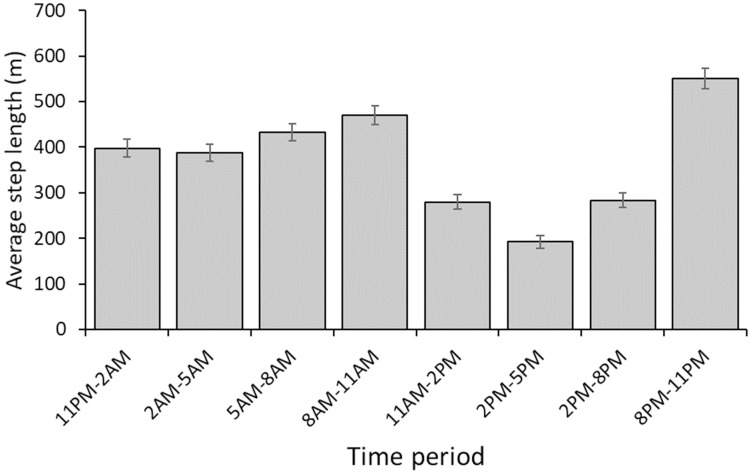
Average three-hour step lengths (with 95% confidence intervals) of GPS-collared wild pigs on Fort Hood Military Installation in 2016–2017.

We used GPS data acquired from 16 adult wild pigs (5 male, 11 female) collared at Fort Hood. Only one individual provided data for analysis through the entire year (i.e., all 4 seasons), but we had data from most individuals for 2–3 seasons, and data for each season from at least nine individuals (Table 1). Average 95% year-round home range was 10.472 ± 0.472 (SE) km^2^. Average winter, spring, summer, and fall 95% home ranges were 8.005 ± 0.611 km^2^, 8.213 ± 0.613 km^2^, 6.720 ± 0.851 km^2^, and 6.150 ± 0.308 km^2^ respectively. We observed no significant difference in 95% home range size by season (F = 0.056, df = 3, *p* = 0.982) or by sex (F = 0.026, df = 1, *p* = 0.8711). Average 50% year-round home range was 1.88 ± 0.116 (SE) km^2^. Average winter, spring, summer, and fall 50% home ranges were 1.420 ± 0.120 km^2^, 1.640 ± 0.137 km^2^, 1.326 ± 0.251 km^2^, and 1.067 ± 0.069 km^2^ respectively. We observed no significant difference in 50% home range size by season (F = 0.009, df = 3, *p* = 0.999) or by sex (F = 0.002, df = 1, *p* = 0.963). Average three-hour step lengths confirmed that the individuals in our study exhibited a crepuscular activity schedule, though activity was greater in the early morning hours than in the evening (Fig 2).

**Fig 3:**
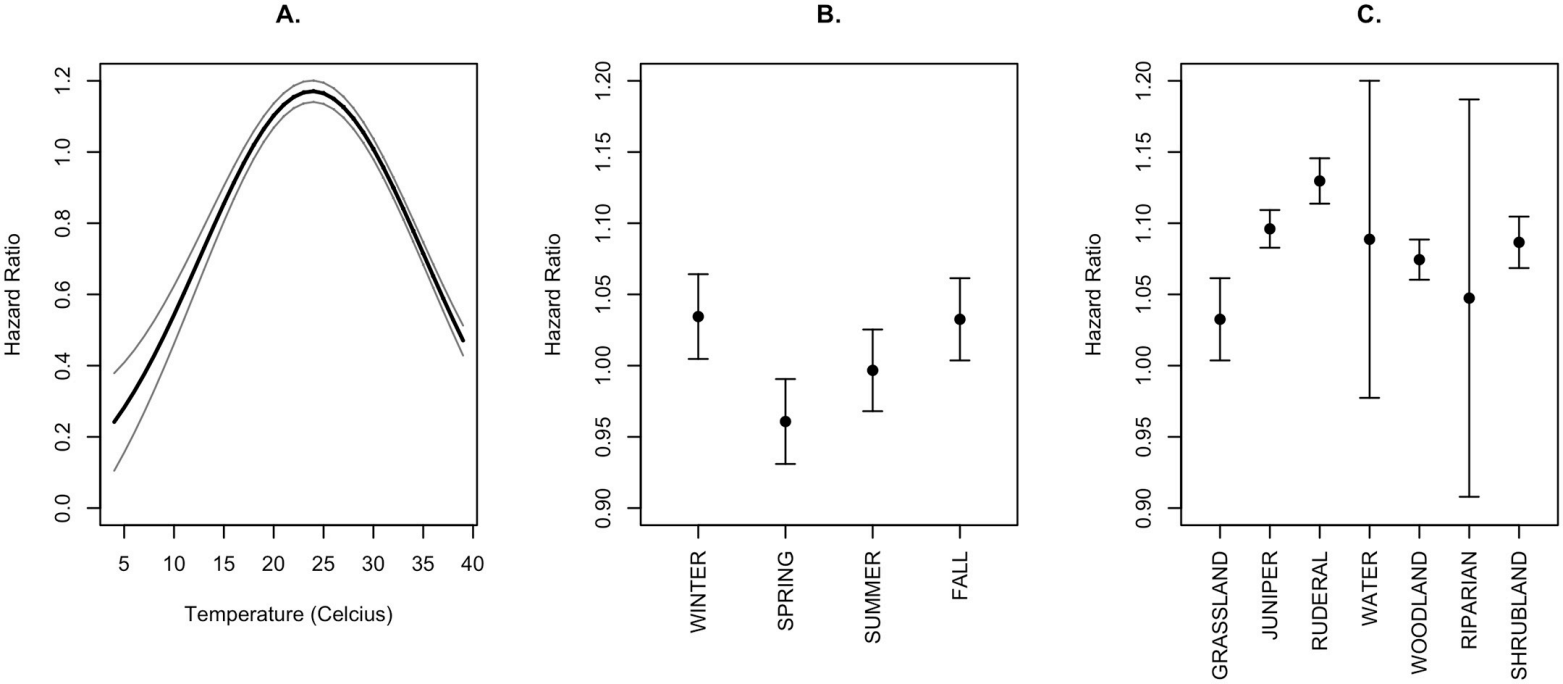
Predicted effects on hazard ratios using our top cox proportional hazard model on A. Temperature, B. Season, and C. Landcover type. 95% confidence intervals are represented by grey lines (A) or horizontal error bars (B, C).

Our top model for variables affecting first passage time was our global model, which held 100% of the akaike weight (Table 3). Within the top model, temperature was the only continuous covariate that had a hazard ratio with a 95% confidence interval that did not overlap one (Table 4). The quadratic form of temperature predicted that hazard ratios were minimized at temperature extremes (Fig 3). Therefore, FPT was longest at the temperature extremes, and pigs were most active when temperatures were moderate (between 15 and 30 °C). The other continuous variables (distance to water and TRI) had 95% confidence intervals that overlapped one and did not affect FPT (Table 4). Both categorical variables (season and land cover) had categories with significant effects on first passage time (Table 3). Pigs were predicted to move more slowly in spring compared to fall and winter, and tended to move at intermediate rates in summer (Fig 3). Pigs were predicted to move slowest in grassland, making it the most used land cover type (Fig 3). Pigs were predicted to move fastest in ruderal areas, making it the least utilized land cover type (Fig 3). Shrubland, woodland, and juniper had similar hazard ratios that fell between grassland and ruderal, and water and riparian areas had large confidence intervals that overlapped one.

**Table 3:** Cox proportional hazard model results relating first passage time of GPS-collared wild pigs on Fort Hood Military Installation, Texas, USA in 2016–2017 to explanatory variables.

	Penalized K^a^	AIC^b^	LL^c^	*wi*^d^
Global	27.96	2812337.92	-1406141	1.00
Temperature^2^	16.96	2812469.92	-1406218	0.00
Season	17.96	2814943.92	-1407454	0.00
Landcover type + distance to water	21.95	2815315.9	-1407636	0.00
Landcover	20.95	2815315.9	-1407637	0.00
Topographic roughness index (TRI)	15.95	2815363.9	-1407666	0.00
Distance to water	15.95	2815367.9	-1407668	0.00

^a^ Penalized degrees of freedom [26]

^b^ Akaike Information Criterion

^c^ Log Likelihood of the model

^d^ Akaike model weight

**Table 4:** Hazard ratios and their associated 95% confidence intervals (CI). Confidence intervals not overlapping 1 indicate an effect of that covariate. Land cover types and seasons are categorical variables where hazard ratios are in reference to the base category. The base landcover category is grassland, and the base season is fall.

Covariate	Hazard ratio (exp^β^)	95% CI
Landcover type: Juniper	1.06	1.03–1.09
Landcover type: Riparian	1.01	0.88–1.17
Landcover type: Ruderal	1.09	1.06–1.13
Landcover type: Shrubland	1.05	1.02–1.09
Landcover type: Water	1.05	0.94–1.18
Landcover type: Woodland	1.04	1.01–1.07
Season: Spring	0.93	0.91–0.95
Season: Summer	0.97	0.95–0.98
Season: Winter	1.00	0.98–1.02
Distance to water	1.00	1.00–1.01
Topographic roughness index	0.99	0.99–1.00
Temperature	0.84	0.83–0.84
Temperature^2^	0.94	0.93–0.94

### Diet

We analyzed stomach contents from 88 wild pigs across four different seasons (winter, n = 23; spring, n = 22; summer, n = 25; autumn, n = 18). A majority of stomach material was plant-based, with each stomach sample possessing at least some plant material (Table 5). However, there was considerable diversity in the types of plants identified, and in support of our hypothesis, consumption of dietary items varied by season. Fruit and soft mass consumption only occurred during spring and summer and was composed of blackberry (*Rubus* spp.) and grape (*Vitis* spp.) species (Table 5). Forb and woody foliage was present in stomachs from all seasons though amounts varied by season (F = 6.168, df = 3, p = 0.001). Specifically, we observed lower volumes of forbs and woody foliage in winter over spring (p = 0.002) and fall (p = 0.003). Hard mast was present in stomachs across each season, primarily consisting of oak (*Quercus* spp.) acorns, as well as occasional hickory (*Carya* spp.) nuts, and honey mesquite (*Prosopis glandulosa*) seed pods during the summer and fall (Table 5). Consumption of hard mast differed between seasons (F = 5.035, df = 3, p = 0.003), and in line with our prediction, less hard mast was consumed in the spring than in the fall (p = 0.032), winter (p = 0.007), and summer (p = 0.008).

**Table 5:** Wild Pig stomach contents. Seasonal variation in stomach contents is expressed as average percent volume (%V) that an item contributed to the total volume on an individual’s stomach (followed by standard error in parentheses), and the frequency (%F) at which an in item was detected at least once in an individual during a season on the Fort Hood Military Installation, Texas, USA, 2016–2017.

Food Category	Winter		Spring		Summer		Fall	
	%V	%F	%V	%F	%V	%F	%V	%F
Plant Matter	62.05 (5.74)	100.00	87.20 (4.18)	100.00	88.89 (2.78)	100.00	71.68 (6.75)	100.00
Graminoids	7.96 (0.02)	95.65	1.20 (0.49)	77.27	1.76 (0.49)	88.00	0.96 (0.32)	61.11
Forb and Woody Foliage	25.96 (4.16)	91.30	73.84 (5.50)	95.45	45.40 (6.55)	92.00	45.41 (6.15)	61.11
Yucca (*Yucca* spp.)	0.14 (0.10)	8.70	9.11 (2.38)	72.73	8.74 (2.03)	84.00	26.77 (4.97)	83.33
Cactus (*Opuntia* spp.)	7.80 (3.03)	39.13	29.17 (6.88)	59.09	10.51 (3.14)	80.00	1.86 (0.94)	33.33
Non-woody Stems	0.00	0.00	6.34 (1.47)	86.36	1.62 (1.31)	20.00	0.13 (0.10)	11.11
Woody Matter	0.58 (0.21)	39.13	1.74 (0.80)	45.45	0.20 (0.13)	12.00	0.09 (0.07)	11.11
Roots/Bulbs/Tubers	0.77 (0.23)	39.13	3.41 (1.58)	59.09	2.67 (2.00)	16.00	0.00	0.00
Corn	7.87 (5.14)	21.74	3.42 (2.41)	18.18	4.34 (2.13)	32.00	11.65 (5.90)	27.78
Unknown Plant Matter	9.68 (2.18)	86.96	15.00 (2.07)	95.45	18.17 (3.15)	100.00	15.20 (3.45)	100.00
Fruits/Soft Mast	0.00	0.00	3.39 (2.54)	22.73	7.15 (3.59)	60.00	0.00	0.00
Blackberries (*Rubus* spp.)	0.00	0.00	3.39 (2.54)	22.73	0.00	0.00	0.00	0.00
Grapes (*Vitis* spp.)	0.00	0.00	0.00	0.00	7.15 (3.59)	60.00	0.00	0.00
Hard Mast	28.13 (5.19)	100.00	8.78 (2.48)	81.82	34.58 (6.49)	84.00	25.32 (5.06)	94.44
Acorns (*Quercus* spp.)	27.78 (5.18)	100.00	8.78 (2.48)	81.82	0.29 (0.11)	32.00	25.12 (5.07)	94.44
Hickory nuts (*Carya* spp.)	0.36 (0.30)	8.70	0.00	0.00	0.01 (0.01)	4.00	0.12 (0.12)	5.56
Honey Mesquite seed pods	0.00	0.00	0.00	0.00	34.28 (6.47)	76.00	0.09 (0.09)	5.56
Animal Matter	19.62 (4.89)	91.30	7.13 (3.07)	72.73	5.22 (2.04)	60.00	7.91 (2.47)	77.78
Insects and Earthworms	12.24 (3.28)	86.96	1.86 (0.69)	59.09	0.26 (0.14)	24.00	4.15 (1.94)	72.22
Spiders	0.00	0.00	0.00	0.00	0.01 (0.01)	4.00	0.00	0.00
Snails	0.31 (0.31)	4.35	0.05 (0.03)	13.64	2.65 (1.19)	40.00	0.33 (0.20)	16.67
Mammals	6.04 (3.94)	13.04	4.90 (3.13)	13.64	2.12 (1.79)	8.00	0.34 (0.34)	5.56
Birds	0.08 (0.08)	4.35	0.00	0.00	0.00	4.00	1.92 (1.34)	16.67
Reptiles/Amphibians	0.50 (0.46)	8.70	0.08 (0.08)	4.55	0.00	0.00	0.00	0.00
Bones	0.11 (0.11)	4.35	0.00	0.00	0.08 (0.07)	8.00	0.00	0.00
Unknown	0.34 (0.21)	13.04	0.24 (0.24)	4.55	0.09 (0.08)	8.00	1.17 (1.14)	11.11
Fungi	2.21 (1.02)	21.74	1.28 (0.70)	22.73	0.30 (0.15)	24.00	8.38 (3.03)	44.44
Lichens	0.00	0.00	0.00	0.00	0.04 (0.04)	4.00	0.00	0.00
Trash	0.55 (0.39)	17.39	0.05 (0.04)	9.09	0.02 (0.02)	4.00	0.22 (0.16)	16.67
Metal	0.00	0.00	0.04 (0.04)	4.55	0.00	0.00	0.00	0.00
Rubber	0.50 (0.39)	13.04	0.01 (0.01)	4.55	0.02 (0.02)	4.00	0.18 (0.16)	11.11
Plastic	0.05 (0.05)	4.35	0.00	0.00	0.00	0.00	0.00	0.00
Clothing fibers	0.00	0.00	0.00	0.00	0.00	0.00	0.04 (0.04)	5.56
Unknown	3.60 (2.08)	30.43	0.49 (0.22)	27.27	0.36 (0.19)	20.00	0.16 (0.11)	11.11
Fibrous Knotted Masses	4.09 (2.71)	17.39	0.43 (0.43)	4.55	0.83 (0.58)	8.00	0.00	0.00

Overall, forbs and woody foliage dominated the vegetative category, followed by graminoids, cactus and yucca (Table 5). Corn was encountered during each season, but at relatively moderate frequencies (20–30% of stomachs) and composed a low proportion of total volume (3–12%). A wide variety of animal matter was detected, although most items outside of insects, earthworms and snails were infrequently encountered (<40% frequency; Table 5). We observed no seasonal differences in vertebrate frequency of occurrence (p = 0.630), but did observe that there tended to be a larger frequency of occurrence of invertebrates in their diet during winter compared to spring and summer (p = 0.002). Fungi were detected in every season (p = 0.070), particularly during the autumn when they were in 44% of all individuals sampled. A small amount of man-made items were discovered in stomachs for each season, primarily consisting of metal, rubber, plastic and clothing fibers (Table 5). We found that the frequency of occurrence of man-made items was larger in the winter and fall compared to other seasons (p = 0.018).

## Discussion

Our findings highlight the benefit of conducting multi-scaled investigations of invasive species behavior to parse out complex resource use patterns. Traditionally, home-range studies are used to assess patterns of resource use [15], but these studies typically only reveal information on the landscape scale that is of minimal value for making targeted management decisions. Indeed, results from our home range analysis indicated that pigs at Fort Hood used large areas throughout the year and failed to capture important finer-scale behavioral patterns that highlighted the opportunistic nature of pig behavior and could be used to focus management. Specifically, results from our finer-scale daily movement and dietary analyses revealed that daily habitat use and movement varied with season, habitat type and temperature, and diet composition also varied with season.

Home range estimates for this study were above the national average for wild pigs (4.92 ± 6.37 km^2^ [11]), though similar to estimates from studies in other arid regions. Large wild pig home ranges were recently reported from an arid grassland in Kent County, Texas (23.9 km^2^, [15]), and in the Chihuahua desert (48.3 ± 4.4 km^2^, and 34.0 ± 4.4 km^2^ for males and females, respectively) and have been attributed to low precipitation amounts [44]. Home range size has been related to precipitation at a global [15] and national scale [36]. Therefore, the large and stable home ranges identified in this study suggest that assessments of pig habitat use at the second order (i.e., selection of location of home ranges) is likely not a useful indicator of pig spatial ecology and potential impacts in this system.

While seasonal and annual wild pig home ranges in our study area were consistently large, there were finer-scale seasonal differences in movement within home ranges (i.e., 3^rd^ order selection). Similar to Kay et al. [27], we found that temperature affected movement on the shorter temporal scale of our first passage times, where intermediate temperatures produced the shortest FPT values, and temperature extremes increased the time spent in one area. These similar results indicate that pigs are most active at intermediate temperatures, which typically occur in the morning and evening and are indicative of crepuscular activity by pigs, as found in other studies [38, 45]. Movement rates of pigs also varied within different land cover types. Our finding that pigs were stationary most frequently in grassland cover types and moved through ruderal areas the quickest suggests that grassland cover was most utilized, ruderal areas were least utilized, and woodland, juniper, and shrubland cover types were of an intermediate use. Our estimates for riparian and water cover types had large confidence intervals, likely due to small sample size of those cover types, and potentially prevented us from being able to interpret their effects.

Preference for remaining in grasslands was counter to our prediction that pigs would prefer cover types that provided cover and water, though grasslands may provide key food resources that are not present in other cover types. Preference for foraging in grasslands has been observed in multiple systems globally, ranging from alpine [46] to coastal grasslands [47]. Similarly, the importance of grasslands as forage is apparent in our diet analysis as graminoids and forbs, the primary grassland vegetation types, were present in most stomachs, especially during the growing season. Further, grasslands were less abundant on the landscape (covering only 10.91% of Fort Hood), collectively suggesting pigs in this system might be following the marginal value theorem [48], where they might remain in grassland cover longer because there is less of a chance of encountering that type again once they leave.

Contrary to other studies [11, 36, 49], first passage time was not affected by distance to water or topographic roughness. The maximum distance to water in our study was 4319 m, but the average was 1046 m. This lack of effect could be because we restricted our analysis to year-round, permanent water sources. It is possible that intermittent water sources were available across the landscape (particularly during certain seasons) and use of these resources could have influenced our results. First passage time was also not significantly affected by topographic roughness index, though contrary to Kim et al. [34], pigs at Fort Hood tended to stay in rougher areas longer. This could be due to the increased effort of traveling across rougher terrain. In Great Smoky Mountains National Park, pigs have been observed to travel up and down slope in response to seasonal food resources [50]. While our study area did not have a similar range in elevations, it is possible that pigs were selecting for microclimates within rougher terrain that provided thermoregulatory or food resource benefits.

Similar to previous studies [51, 52], pig diet at Fort Hood was primarily plant-based. While we did not sample availability of food items, seasonal availability of plant food items likely influenced pig diet [23, 43], where hard mast occurred less in spring and soft mast occurred less in winter. By contrast, animal matter increased in winter to above the average amount for wild pigs as reported by Ballari and Barios-Garcia [51]. This increase of animal matter in the diet could indicate that plant food availability was most limited during winter, or that animal matter provided an important source of protein in colder winter months [51]. Similar to pigs in coastal South Carolina, fungi occurred in stomachs from all seasons at a low volume, though, at Fort Hood, fungi consumption peaked in the fall, whereas in South Carolina, fungi consumption peaked in summer [22]. Lastly, even though all stomach analysis in this study were completed by one observer, it is important to note that there will always be potential for bias in stomach analysis methods due to differential digestion rates of food types [51].

Collectively, by combining our diet and movement results we gained a more nuanced understanding of behavioral patterns of pigs that can be used to guide future ecological impact assessments and where and when to focus control efforts. By incorporating information about daily movement, we were able to find that grassland cover types should be targeted in removal efforts due to their open cover and preference by wild pigs on Fort Hood. The addition of diet analysis suggested vegetative food items composed a large portion of the diet, but were least commonly ingested during the winter season. Thus, while multiple factors need to be considered while trapping including group size and ease of access for placing and checking traps, baited trapping in grasslands during the winter is likely to be the most productive for capturing pigs. Animal matter increased in pig diets during winter and consisted mostly of insects and earthworms that would be found while rooting through soil. In addition, pigs moved more quickly in winter and fall than the spring, suggesting that foraging for animal matter is likely to cause a higher rate of soil disturbance across wide areas during winter. By contrast, foraging in other seasons was likely to have the highest impact on vegetation. Spring forbs may be particularly at risk since pigs ingested forbs at a high rate in spring and moved more slowly, possibly indicating that when a forb patch is found, pigs remain in that patch to eat their fill.

Implementing our multi-scale approach in other environments that wild pigs occur in, or in studies of other invasive species, could inform site- and species-specific monitoring and management. A similar study could better focus control efforts of wild pigs in the southeast coastal plain where the landscape of pine forests, agriculture, and developed areas is becoming more fragmented [53] and pigs are likely to use resources differently than in Texas. Also, since wild pigs learn to avoid control measures over time [54], a multi scaled approach could help determine the continual effectiveness of a management action by analyzing wild pig movement and diet for shifts in behavior due to control evasion. Further, continued multi-scale studies have the potential to expose more complicated relationships that are helpful in understanding invasive species ecology and the response of ecosystems [55]. This greater biological understanding can then help managers make more focused and informed decisions on species control and impact mitigation.
